# Relationship between symptoms, sociodemographic factors, and general practice help-seeking in 10 904 adults aged 50 and over

**DOI:** 10.1093/eurpub/ckae198

**Published:** 2024-12-15

**Authors:** Rosalind Adam, Rute Vieira, Philip C Hannaford, Kathryn Martin, Katriina L Whitaker, Peter Murchie, Alison M Elliott

**Affiliations:** Institute of Applied Health Sciences, University of Aberdeen, Aberdeen, United Kingdom; Institute of Applied Health Sciences, University of Aberdeen, Aberdeen, United Kingdom; Institute of Applied Health Sciences, University of Aberdeen, Aberdeen, United Kingdom; Institute of Applied Health Sciences, University of Aberdeen, Aberdeen, United Kingdom; School of Health Sciences, University of Surrey, Surrey, United Kingdom; Institute of Applied Health Sciences, University of Aberdeen, Aberdeen, United Kingdom; Graduate School, Abertay University, Dundee, United Kingdom

## Abstract

Symptoms are a common reason for contact with primary care. This study investigated associations between symptom-related, demographic, social, and economic factors on general practice (GP) help-seeking. Secondary analysis of responses to a 25-symptom questionnaire, from 10 904 adults aged ≥50 years reporting at least one symptom in the preceding year. Cluster analysis and univariable and multivariable logistic regressions explored associations between self-reported GP help-seeking, symptom-related factors, and respondent characteristics. Most respondents, 7638 (70%), reported more than one symptom in the preceding year. Ten symptom clusters were identified. Most included common symptoms like headache and back or joint pain. There were increased odds of help-seeking in females, those with poorer health status and those unable to work due to illness/disability when multiple symptoms were reported, but not when single symptoms were reported. Age and sex had variable effects on help-seeking, depending on the symptom. Reporting poorer health status, more comorbidities, and being unable to work due to illness or disability increased odds of help-seeking across a diverse variety of symptoms. Single people and those reporting lower social contact had lower odds of help-seeking for some symptoms. Being a current smoker reduced odds of help-seeking for persistent indigestion/heartburn, persistent cough, coughing up phlegm, and shortness of breath. Factors associated with self-reported help-seeking vary for different symptoms. Poorer health and adverse economic and social factors are associated with increased GP help-seeking. These wider determinants of health interact with symptom experiences and will influence GP workload.

## Introduction

Primary care systems throughout the world face service delivery and access challenges [1]. General practice (GP) in the United Kingdom (UK) is under pressure to deliver more in-person appointments than ever before, with fewer doctors [2]. English GPs delivered 34.3 million appointments in October 2023 compared to 30.8 million in October 2019 [3]. Despite this, patients report difficulties accessing primary care [4]. It is crucial to understand factors that influence decisions to seek help from GP.

Many researchers and policy makers have focused on the challenges of ageing and multimorbidity as drivers of GP workload [5]. However, many patients present to GP with symptoms rather than defined diseases [6]. Most symptoms experienced are never presented to a medical professional, and of those that are, most do not result in disease diagnosis (the ‘symptom iceberg’) [6, 7].

Help-seeking for symptoms has been investigated at the level of the individual [8]. Research points to an interplay between biomedical, psychological, social, and cultural factors that influence symptom appraisal and decisions to seek help [9, 10]. Factors that might influence help-seeking at the population level are less well understood. For example, a recent systematic review found that older age was likely to be an important modifier of symptom appraisal and help-seeking for potential cancer symptoms [11]. However, the direction of association was unclear with some studies suggesting a longer patient interval (time between symptom awareness and seeking help) in older age, and others indicating a shorter patient interval [11].

Most research so far has considered help-seeking for single symptoms. For example, in a study of 2000 people with cancer, those with a lump sought help more quickly than those who had unexplained weight loss or a change in bowel or bladder habit [12]. As well as the nature of single symptoms, help-seeking might be influenced by patterns of symptoms occurring within a certain time frame. Little is known about how symptoms might cluster in community-dwelling adults, or about how symptom clusters might influence help-seeking.

This study used data from a large community-based questionnaire study in adults aged 50 and over, to investigate associations between symptom-related, sociodemographic factors and GP help-seeking. Symptom-related factors of interest included single or multiple symptoms, symptom clusters (a specified combination of more than one symptom reported as having occurred within the same 12-month period), and previous experience of the symptom. Understanding patterns of symptoms in the community and factors associated with help-seeking could give important insights into whether there are predictable and modifiable determinants of primary care workload. Another aim was to identify individuals who may be less likely to seek help for symptoms and possibly at risk of poorer health outcomes.

## Methods

### Data and data-handling

This was a secondary analysis of data from the Understanding Symptom Experiences Fully (USEFUL) questionnaire study [13]. USEFUL was conducted between 2015 and 2016. It asked about the frequency of, and help-seeking responses to 25 symptoms including those associated with four common cancers (see Box 1). Questionnaire recipients were told that the study was enquiring about symptoms experienced, their effects, thoughts about the symptoms, and actions taken. Cancer was not mentioned in any participant-facing material.

Box 1. Symptoms enquired about in the Understanding Symptom Experiences Fully (USEFUL) questionnaire (listed in the same order as presented in the questionnaire)

**Table:** 

Headaches
Persistent indigestion/heartburn
Difficulty swallowing
Stomach or abdominal pain
Chest pain
Hoarseness
Loss of appetite
Unexplained weight loss
Persistent cough
Change in ongoing cough
Persistent diarrhoea
Persistent constipation
Coughing up phlegm
Coughing up blood
Shortness of breath
Wheezy chest
Change in bladder habits
Change in bowel habits
Blood in stool or rectal bleeding
Back or joint pain
Persistent vomiting
Vomiting up blood
Lump in breast
Breast change other than lump
Tired all the time

Data about the respondent’s experience of each symptom in the preceding 12 months, whether the respondent had contacted their GP about each symptom (yes/no), and whether the symptom had been experienced before the last year were collected. The questionnaire sought demographic (sex, age, marital status), current health (self-reported health status, co-morbidities, smoking status), socioeconomic (employment, household income, education, social contact), and GP-related (practice location, distance, and travelling time to GP) information [13].

The questionnaire was sent to a random sample of 50 000 adults aged ≥50 years, registered at 21 GPs across Scotland and the West Midlands of England. Non-respondents received reminders 3 and 6 weeks after the initial mailing. Questionnaires could be completed online or by post.

Data from respondents were included *if* there was a report of having experienced at least one of the 25 symptoms within the preceding 12 months *and* a response about whether they contacted their GP about at least one symptom (yes/no). A study flowchart is included in Supplementary Data File 1.

#### Characterizing the sample and exploring symptom clusters

Descriptive statistics were used to summarize overall and relevant subgroup information regarding each symptom, previous experience of the symptom, GP help-seeking behaviour, and the characteristics of respondents. Categorical variables were expressed as frequencies (percentage) and continuous variables as median (range).

Respondents were grouped according to their experience of single symptoms (if only one symptom reported) or symptom clusters (if more than one symptom reported). Cluster analysis using partitioning cluster methods identified distinct patterns of symptoms experienced within the 1-year period [14]. Respondents reporting a combination of headaches and back or joint pain (BJP), but no other symptoms, were clustered *a priori*, as these were the most common symptoms to be reported alone, as well as combined with other symptoms. This reduced the influence of this cluster when identifying others. Every participant who experienced more than one symptom contributed to a cluster. All groups were mutually exclusive; individuals could only be in one cluster. Supplementary Data File 2 gives additional methodological details regarding the cluster analysis.

#### Exploring factors associated with GP help-seeking

The main outcome was dichotomous: ‘contacted GP’ or ‘did not contact GP’. Independent univariable and multivariable logistic regressions were used to explore associations between GP help-seeking and socio-demographic characteristics within those individuals with (i) pre-existing symptoms (more than 12 months), (ii) symptoms experienced only in the last year, (iii) single symptoms experienced within the past year, and (iv) multiple symptoms experienced in the past year. Independent univariable and multivariable regressions were also used to explore the association between help-seeking, the socio-demographic characteristics and symptom clusters for each symptom experienced.

Models were built independently for each symptom, using only data from the respondents that reported that specific symptom, alone or with other symptoms. Therefore, the groups of respondents included in each analysis were mostly distinct. A complete case data analysis was performed.

A two-stage strategy was used to build the final adjusted models. In the first step, univariable logistic regression models were estimated for each independent variable, and the statistical significance of the resulting unadjusted odds ratio (OR) was assessed at *α* = 0.05. Secondly, all independent variables that were statistically associated (*P* < 0.05) with the outcome in the univariable analyses were included in the initial multivariable model. To obtain the most parsimonious and informative multivariable model, we used the Akaike Information Criteria (AIC) which estimates the relative information lost by a given model, as the criteria for retaining (if AIC is at least two units lower) or removing predictors from the final model [15]. Goodness-of-fit was assessed using the Hosmer–Lemeshow test and multicollinearity was explored using variation inflation factors (VIFs) [16, 17].

## Results

### Sample characteristics

Of 50 000 posted questionnaires, 16 778 questionnaires were returned (response 33.6%), from which 3357 (20%) reported having no symptoms, and 2517 (15%) did not provide information on GP help-seeking when they reported a symptom (see flowchart, Supplementary Data File 1). Therefore, 10 904 (65%) respondents were included in the analysis.

Respondent characteristics are summarized in Table 1. Male and female patients were reasonably evenly represented (45.7% versus 54.3%, respectively). Most respondents were aged between 50 and 70 years (73.4%), married/partnered (74.3%), rated their health as either excellent, very good or good (81.1%), and reported 1 or 2 co-morbidities (54.5%); a substantial minority reported 3 or more co-morbidities (24.3%).

**Table 1.** Demographics of questionnaire respondents included in this study (*n* = 10 904)

Demographic group	Sub-group	*n*	%
Sex	Male	4983	45.7
	Female	5922	54.3
Age group	50–59	3857	35.4
	60–69	4140	38.0
	70–79	2263	20.8
	80+	644	5.9
Marital status	Single	704	6.5
	Married/living together	8106	74.3
	No longer married	1959	18.0
	Missing	135	1.2
Health status	Excellent	1026	9.4
	Very good	3824	35.1
	Good	3991	36.6
	Fair	1558	14.3
	Poor	329	3.0
	Missing	176	1.6
Comorbid conditions^a^	None	2318	21.3
	1 or 2	5938	54.5
	3 or more	2648	24.3
Smoking status	Never smoked	5814	53.3
	Ex-smoker	4008	36.8
	Current smoker	909	8.3
	Missing	173	1.6
Employment status	Working full-time	2609	23.9
	Working part-time	1091	10.0
	Self-employed	834	7.7
	Retired	5562	51.0
	Unable to work due to illness/disability	270	2.5
	Others not in paid employment	379	3.5
	Missing	159	1.5
Household income	<£15 000	2200	20.2
	£15 000–29 999	2955	27.1
	£30 000–49 999	2471	22.7
	>£500 000	2127	19.5
	Missing	1151	10.6
Educational status	No educational qualifications	1183	10.8
	Secondary school or equivalent	3466	31.8
	College/vocational courses and other	424	3.9
	Professional qualification	2900	26.6
	Degree or postgraduate qualification	2732	25.1
	Missing	199	1.8
Level of social contact	Low	609	5.6
	Medium	3659	33.6
	High	6115	56.1
	Missing	521	4.8
Country of general practice registration	Scotland	5728	52.5
	England	5176	47.5
Distance from general practice	Under 1 mile	3564	32.7
	1–5 miles	4940	45.3
	6–10 miles	542	5.0
	Missing	1858	17.0
Travel time to general practice	<15 min	6919	63.5
	15–29 min	1846	16.9
	30–59 min	261	2.4
	Missing	1878	17.2

a Ever been diagnosed with asthma, cancer, epilepsy, chronic bronchitis/COPD, other chest disorders, heart disorder, stroke, diabetes, high blood pressure, liver disorder, arthritis/rheumatic disorder, mental health disorder, thyroid disorder, stomach/digestive disorder, other condition (to be specified).

### Descriptive results: symptoms experienced, GP help-seeking, and previous experience of the symptom

Most respondents (*n* = 7638, 70%) had experienced more than one symptom in the preceding 12 months (range 1–18, median 2).

The most common symptoms reported were back or joint pain (BJP) (*n* = 6809, 62.4%), headaches (*n* = 4997, 45.8%), feeling tired all the time (*n* = 2155, 19.8%), and persistent indigestion/heartburn (*n* = 2072, 19.0%) (see Table 2). For all symptoms except headaches and BJP, over half of the respondents reported experiencing at least three other symptoms within the preceding 12 months (see Supplementary Data File 3). Headaches and BJP were also most frequently experienced alone during the preceding year.

**Table 2.** Symptoms experienced at least once in the preceding year, GP help-seeking, and presence of symptom before last year among individuals in the sample (*N* = 10 904)

	All (10 904) respondents who had at least one symptom and indicated whether they had consulted their GP about that symptom				Those with single symptom only (3266)			
Symptom	Individuals reporting symptoms within last year (number, %)	**Individuals reporting the same symptom also experienced before the last year (number, %)** ^a^	GP contacted within last year (number, %)	Individuals consulting who had also experienced the symptom before the last year (number, %)	Individuals reporting symptoms without other symptoms within last year (number, %)	**Individuals reporting the single symptom also experienced before the last year (number, %)** ^a^	GP contacted within last year number, % of those reporting that single symptom)	Individuals consulting who had also experienced the single symptom before the last year (number, %)
Upper gastrointestinal symptoms								
Persistent indigestion/heartburn	2072 (19.0)	1665/2014 (82.7)	957 (46.2)	762 (79.6)	161 (4.9)	130/159 (81.7)	69 (42.9)	55 (79.7)
Difficulty swallowing	606 (5.6)	377/589 (64.0)	237 (39.1)	140 (59.1)	21 (0.6)	13/21 (61.9)	11 (52.4)	7 (63.6)
Stomach or abdominal pain	2032 (18.6)	1517/1960 (77.4)	1038 (51.1)	743 (71.6)	105 (3.2)	68/103 (66.0)	72 (68.6)	46 (63.9)
Persistent vomiting	48 (0.4)	26/45 (57.8)	36 (75.0)	22 (61.1)	2 (0.1)	0/2 (0.0)	1 (50.0)	0 (0.0)
Vomiting up blood	6 (0.1)	1/6 (16.7)	4 (66.7)	0 (0.0)	0 (0.0)	0/0 (0.0)	0 (0.0)	0
Respiratory or cardiovascular symptoms								
Chest pain	1014 (9.3)	666/971 (68.6)	626 (61.7)	387 (61.8)	42 (1.3)	18/39 (46.2)	31 (73.8)	10 (32.3)
Hoarseness	921 (8.4)	586/870 (67.4)	267 (29.0)	160 (59.9)	26 (0.8)	12/22 (54.5)	7 (26.9)	3 (42.9)
Persistent cough	1529 (14.0)	947/1470 (64.4)	821 (53.7)	507 (61.8)	109 (3.3)	57/103 (55.3)	72 (66.1)	37 (51.4)
Change in ongoing cough	147 (1.3)	82/133 (61.7)	96 (65.3)	55 (57.3)	3 (0.1)	3/3 (100.0)	0 (0.0)	0 (0.0)
Coughing up phlegm	1599 (14.7)	1235/1537 (80.4)	628 (39.3)	466 (74.2)	86 (2.6)	67/85 (78.8)	40 (34.9)	23 (57.5)
Coughing up blood	50 (0.5)	25/49 (51.0)	38 (76.0)	18 (47.4)	2 (0.1)	1/2 (50.0)	2 (100.0)	1 (50.0)
Shortness of breath	1823 (16.7)	1365/1744 (78.3)	1051 (57.7)	769 (73.2)	91 (2.8)	54/88 (61.4)	63 (69.2)	34 (54.0)
Wheezy chest	1206 (11.1)	966/1152 (83.9)	699 (58.0)	553 (76.3)	48 (1.5)	38/45 (84.4)	29 (60.4)	22 (75.9)
Colorectal symptoms								
Persistent diarrhoea	489 (4.6)	331/477 (69.4)	252 (51.5)	155 (61.5)	24 (0.7)	12/24 (50.0)	19 (79.2)	7 (36.8)
Persistent constipation	644 (5.9)	502/612 (82.0)	319 (49.5)	236 (74.0)	33 (1.0)	29/31 (93.5)	17 (51.5)	14 (82.4)
Change in bowel habits	854 (7.8)	504/819 (61.5)	457 (53.5)	279 (61.1)	45 (1.4)	27/44 (61.4)	27 (60.0)	15 (55.6)
Blood in stool or rectal bleeding	656 (6.0)	453/659 (68.7)	316 (48.2)	192 (60.8)	59 (1.8)	45/59 (76.3)	24 (40.7)	19 (79.2)
Breast symptoms								
Lump in breast	113 (1.0)	55/107 (51.4)	99 (87.6)	47 (47.5)	18 (0.6)	4/17 (23.5)	17 (94.4)	4 (23.5)
Breast change other than lump	139 (1.3)	63/135 (46.7)	106 (76.3)	45 (42.5)	14 (0.4)	6/14 (42.9)	10 (71.4)	3 (30.0)
Other symptoms								
Loss of appetite	582 (5.3)	302/549 (55.0)	196 (33.7)	106 (54.1)	13 (0.4)	6/13 (46.2)	5 (38.4)	3 (60.0)
Unexplained weight loss	199 (1.8)	65/182 (35.7)	131 (65.8)	50 (38.2)	3 (0.1)	1/3 (33.3)	3 (100)	1 (33.3)
Tired all the time	2155 (19.8)	1533/2077 (73.8)	976 (45.3)	710 (72.7)	84 (2.6)	61/83 (73.5)	36 (42.9)	24 (66.7)
Headaches	4997 (45.8)	4277/4851 (88.2)	803 (16.1)	618 (77.0)	614 (18.8)	526/604 (87.1)	91 (14.8)	63 (69.2)
Change in bladder habits	1219 (11.2)	700/1169 (59.9)	703 (42.3)	43 (61.6)	141 (4.3)	71/136 (52.2)	103 (73.0)	48 (46.6)
Back or joint pain	6809 (62.4)	5983/6666 (89.8)	3165 (46.5)	2746 (86.8)	1522 (46.6)	1295/1500 (86.3)	741 (48.7)	593 (80.0)

a Number of individuals who answered the question. Participants were asked to indicate ‘yes’ or ‘no’ to indicate whether they had experienced the symptom before the last year. Those that were left blank were missing data, hence the denominator may be lower than the total number of individuals reporting the symptom in the past year.

Many of the respondents who experienced a symptom within the preceding year had also experienced this symptom more than a year previously although the proportion varied for different symptoms (see Table 2).

The proportion of respondents who reported that they had contacted their GP for each symptom varied widely. For example, 99 out of 113 (87.6%) sought help for a lump in the breast, whereas 803 out of 4997 (16.1%) sought help for headache (Table 2). The proportion of those who sought help for a symptom who had also experienced the symptom prior to the last year varied by symptom. For example, 2747 out of the 3165 (86.8%) respondents who had contacted their GP about back pain had also experienced back pain prior to the last year whereas none of the four participants who had contacted their GP about vomiting blood had experienced that symptom before.

### Symptom clusters

After *a priori* clustering of headaches and BJP, 10 other multi-symptom clusters were identified in the cluster analysis. These clusters are described in Table 3 including the number and percentage of respondents in each cluster who experienced each symptom. The most prominent symptoms across all clusters were BJP (present in more than 50% of individuals in 8 of the 10 clusters) and headache (present in more than 50% of individuals in six clusters). Every cluster included a pain symptom (headache, BJP, chest pain, or stomach or abdominal pain).

**Table 3.** Symptom clusters derived from 7638 individuals with more than one symptom

	Percentage of individuals in the cluster reporting each symptom (denominator is number in that cluster)																								
	Headaches	Persistent indigestion/heartburn	Difficulty swallowing	Stomach or abdominal pain	Chest pain	Hoarseness	Loss of appetite	Unexplained weight loss	Persistent cough	Change in ongoing cough	Persistent diarrhoea	Persistent constipation	Coughing up phlegm	Coughing up blood	Shortness of breath	Wheezy chest	Change in bladder habits	Change in bowel habits	Blood in stool or rectal bleeding	Back or joint pain (BJP)	Persistent vomiting	Vomiting up blood	Lump in breast	Breast change other than lump	Tired all the time
Cluster and number (%/7638) of individuals in the cluster																									
Headaches and back or joint pain (BJP) cluster (*n* = 733, 9.6%)	**100**	0	0	0	0	0	0	0	0	0	0	0	0	0	0	0	0	0	0	**100**	0	0	0	0	0
Abdominal pain,^a^ headaches and BJP cluster (*n* = 802, 10.5%)	**75**	0	7	**100**	12	9	9	2	9	1	9	11	9	1	6	4	10	18	9	**81**	1	0	1	2	24
Indigestion, headaches, and BJP cluster (*n* = 1221, 16.0%)	**80**	**75**	7	11	11	11	5	2	8	1	6	8	8	0	11	9	6	8	7	**85**	1	0	1	2	12
Persistent cough and headaches cluster (*n* = 668, 8.7%)	**84**	4	7	3	7	12	6	2	**57**	3	4	6	7	1	16	14	15	7	11	12	0	0	1	2	6
Tiredness, headaches and BJP cluster (*n* = 712, 9.3%))	**69**	0	6	0	9	8	9	4	6	0	4	7	6	0	12	3	9	7	6	**74**	0	0	2	2	**100**
Coughing phlegm and BJP cluster (*n* = 1231, 16.1%)	18	9	9	6	8	16	5	3	27	2	5	8	**67**	1	11	10	7	11	10	**68**	0	0	1	2	8
Cluster																									
Varied multi-symptom cluster (*n* = 338, 4.4%)	**89**	**80**	20	**85**	27	21	25	5	15	3	20	24	12	1	**70**	15	27	24	12	**93**	3	0	3	3	**93**
Shortness of breath, wheezy chest, and BJP cluster (*n* = 686, 9.0%)	10	6	6	8	16	14	7	3	15	3	4	4	17	1	**86**	**62**	5	5	6	**67**	0	0	0	1	20
Indigestion and abdominal pain cluster (*n* = 549, 7.2%)	13	**74**	9	**73**	16	9	8	3	15	0	11	11	3	0	13	7	15	17	10	17	1	0	2	1	23
Bladder symptom and BJP cluster (*n* = 382, 5.0%)	20	10	8	5	7	6	4	3	4	0	5	10	0	0	16	2	**100**	21	10	**79**	0	0	1	2	20
Respiratory predominant multi-symptom cluster (*n* = 316, 4.1%)	**84**	33	19	45	**66**	35	22	6	**78**	13	9	10	**78**	3	**88**	**80**	22	15	9	**81**	2	1	3	3	**70**

a Abdominal pain was asked about as ‘stomach or abdominal pain’ in the original questionnaire and has been abbreviated to abdominal pain in the clusters for presentation purposes.

Symptoms experienced by at least 50% of individuals in the cluster are highlighted in yellow and bold.

Five clusters contained disparate symptoms that did not seem to relate to a particular body system. Five clusters contained symptoms that might fit predominantly within a body system. An indigestion and abdominal pain cluster was identified, which encompassed symptoms that predominantly related to the gastrointestinal system. Similarly, a respiratory symptom cluster contained multiple respiratory symptoms. ‘Back or joint pain’ was asked about together in the questionnaire. The bladder symptom and BJP cluster could relate predominantly to the urological/renal system. The coughing phlegm and BJP and the shortness of breath, wheezy chest, and BJP clusters could predominantly relate to the respiratory system.

### Factors associated with GP help-seeking

Results from multivariable regressions investigating odds of help-seeking across the demographic, socioeconomic and health-related factors are presented in Table 4. Univariable results are in Supplementary Data File 4. Univariable and multivariable models were also conducted for each different symptom. Statistically significant results from the symptoms models are summarized in Supplementary Data File 5 and full results for the univariable and multivariable symptoms models are in Supplementary Data Files 6 and 7, respectively.

**Table 4.** Odds of GP help-seeking for single and multiple symptoms, new and pre-existing symptoms across a range of sociodemographic factors

Sociodemographic factor	Odds of seeking help: single symptom^a^	Odds of seeking help: multiple symptoms^a^	Odds of seeking help, new symptom^b^	Odds of seeking help, pre-existing symptom^b^
Male sex	Reference category			
Female sex	–	1.40 (1.24–1.57)	–	1.04 (0.87–1.26)
Age group 50–59	Reference category			
Age group 60–69	1.17 (0.89–1.54)	1.15 (0.98–1.36)	1.09 (0.956–1.24)	0.73 (0.56–0.94)
Age group 70–79	1.49 (1.05–2.11)	1.18 (0.95–1.47)	1.21 (1.02–1.44)	0.57 (0.41–0.80)
Age group 80+	1.63 (1.01–2.65)	1.65 (1.19–2.32)	1.59 (1.21–2.08)	0.96 (0.62–1.47)
Marital status: married/living together	Reference category			
Marital status: single	–	0.78 (0.62–0.98)	0.78 (0.64–0.94)	0.75 (0.52–1.06)
No longer married	–	0.95 (0.81–1.12)	1.07 (0.94–1.22)	1.05 (0.83–1.34)
Health status: excellent	Reference category			
Health status: very good	1.43 (1.06–1.95)	1.47 (1.17–1.86)	1.64 (1.39–1.94)	1.38 (1.06–1.80)
Health status: good	2.17 (1.58–3.00)	1.57 (1.25–1.99)	2.18 (1.84–2.58)	1.58 (1.19–2.15)
Health status: fair	2.41 (1.55–3.78)	2.78 (2.12–3.64)	3.96 (3.21–4.89)	2.78 (1.86–4.16
Health status: poor	1.96 (0.71–5.38)	5.31 (3.23–9.05)	6.42 (4.23–10.04)	5.81 (2.48–13.55)
Comorbid conditions: none	Reference category			
Comorbid conditions:	1.61 (1.27–2.06)	1.70 (1.46–1.98)	1.68 (1.49–1.89)	1.44 (1.16–1.78)
1 or 2				
Comorbid conditions:	2.06 (1.47–2.87)	2.78 (2.30–3.36)	2.99 (2.56–3.49)	1.94 (1.42–2.64)
3 or more				
Never smoked	Reference category			
Ex-smoker	–	1.05 (0.93–1.19)	–	1.02 (0.84–1.23)
Current smoker	–	0.90 (0.73–1.10)	–	0.76 (0.54–1.07)
Working full-time	Reference category			
Working part-time	0.97 (0.68–1.39)	1.09 (0.88–1.35)	1.14 (0.96–1.35)	1.02 (0.71–1.44)
Self-employed	0.96 (0.63–1.45)	1.00 (0.81–1.25)	0.95 (0.79–1.14)	1.36 (0.95–1.93)
Retired	0.86 (0.62–1.18)	0.95 (0.78–1.14)	0.92 (0.79–1.07)	1.12 (0.83–1.52)
Unable to work due to illness/disability	0.91 (0.35–2.37)	1.83 (1.15–3.01)	1.99 (1.31–3.13)	1.59 (0.61–3.92)
Others not in paid employment	0.93 (0.51–1.69)	1.37 (0.98–1.93)	1.22 (0.94–1.59)	1.26 (0.71–2.15)
Household income >£50 0000	Reference category			
Household income < £15 000	1.10 (0.79–1.54)	1.16 (0.94–1.43)	1.15 (0.98–1.35)	1.81 (1.29–2.54)
Household income £15 000–29 999	0.95 (0.71–1.27)	1.20 (1.01–1.43)	1.07 (0.93–1.22)	1.49 (1.12–1.98)
Household income £30 000–49 999	1.01 (0.76–1.35)	1.16 (0.99–1.37)	1.09 (0.95–1.24)	1.25 (0.94–1.65)
Degree or postgraduate qualifications	Reference category			
No educational qualifications	0.91 (0.63–1.31)	1.16 (0.92–1.46)	–	0.79 (0.57–1.10)
Secondary school or equivalent	0.92 (0.71–1.20)	1.14 (0.97–1.33)	–	1.01 (0.79–1.28)
College/vocational courses and other	0.94 (0.53–1.66)	1.25 (0.92–1.71)	–	0.74 (0.43–1.22)
Professional qualification	0.82 (0.63–1.06)	1.07 (0.92–1.25)	–	1.04 (0.81–1.33)
Distance to GP under one mile	Reference category			
Distance to GP 1–5 miles	0.90 (0.74–1.09)	–	–	–
Distance to GP 6–10 miles	0.58 (0.37–0.90)	–	–	–

a Odds of seeking help for a single symptom is for those individuals who only reported one symptom in isolation within the previous 12 months. Multiple symptoms are those who experienced more than one symptom within the previous 12 months.

b Odds of seeking help for a new symptom refers to those who experienced a single symptom as a new symptom within the past 12 months. Pre-existing symptoms refer to those single symptoms that were present prior to the past 12 months.

The table shows odds ratios with 95% confidence intervals. Only factors that reached statistical significance in the univariable analysis are shown here (full results in Supplementary Data File 4).

The sociodemographic factors that did not have a significant association with help-seeking in the univariable models were: social contact, GP registration in Scotland/England, and travel time to GP. These have been omitted from this table for conciseness, but the full univariable and multivariable results are in Supplementary Data File 4.

### Sex, age, and GP help-seeking

Females had increased odds of help-seeking when multiple symptoms were experienced compared to males. In the symptoms models, females (compared to males) were more likely to seek help for unexplained weight loss and changes in bladder habits. However, females had lower odds of help-seeking for six symptoms: headache, chest pain, persistent diarrhoea, shortness of breath, blood in stool/rectal bleeding, and BJP.

Compared to respondents in the 50–59-year age group, those in the 80+ age group had higher odds of help-seeking for single symptoms, multiple symptoms and new symptoms, but not for pre-existing symptoms. In fact, those in the 60–69- and 70–79-year age groups had lower odds of seeking help for pre-existing symptoms compared to those in the 50–59-year age group. The symptoms models showed that increasing age was associated with increased help-seeking for persistent diarrhoea and BJP (70–79-year age group) but lower odds of help-seeking in the 60–69- and 70–79-year age groups for headaches.

### Health status, comorbidities, and smoking

Poorer self-rated general health status was associated with increased odds of help-seeking (see Table 4). For example, those who experienced multiple symptoms and rated their health status as poor had over five times the odds of seeking help than those who rated their health as excellent (see Table 4). In the symptoms model, poorer health status was associated with increased help-seeking for BJP, shortness of breath, persistent diarrhoea, and headaches.

Compared to having no comorbid conditions, those with 1 or 2 comorbidities had increased odds of seeking help for single, multiple, new, and pre-existing symptoms (similar effect sizes, see Table 4). The odds of seeking help were further increased for those with 3+ comorbid conditions. This effect was also observed in the symptoms models, with comorbid conditions increasing the odds of help-seeking for eight diverse symptoms (see Supplementary Data File 5).

Smoking status did not have a significant effect on odds of help-seeking in general, however in the symptoms models, being a current smoker (compared to a never smoker) was associated with lower odds of help-seeking for persistent indigestion/heartburn, persistent cough, coughing up phlegm, and shortness of breath.

### Social and economic factors

Compared to being married/living together, respondents who were single had lower odds of help-seeking for multiple symptoms and new symptoms. In the symptoms analyses, being single was associated with lower odds of help-seeking for shortness of breath. In the overall analysis (Table 4), social contact was not associated with help-seeking. However, in the symptoms model, ‘medium’ or lower social contact (compared to high social contact) was associated with reduced odds of help-seeking for eight symptoms: headaches, BJP, stomach or abdominal pain, chest pain, loss of appetite, shortness of breath, wheezy chest, and feeling tired all the time (see Supplementary Data File 5).

Compared to working full-time, being unable to work due to illness/disability was associated with increased odds of help-seeking for those with multiple symptoms, new symptoms, and pre-existing symptoms (but not single symptoms) (Table 4). Being unable to work increased the odds of help-seeking for headache, persistent indigestion, wheezy chest, blood in stool/rectal bleeding, and BJP. Being self-employed reduced the odds of help-seeking for persistent indigestion/heartburn and change in bowel habits.

Household income and educational status did not have a pronounced effect on help-seeking overall (Table 4). However, in the symptoms model, it was evident that lower household income was associated with increased odds of help-seeking for headaches, shortness of breath, and feeling tired all the time. Similarly, those with fewer educational qualifications had higher odds of help-seeking for difficulty swallowing and change in bowel habits.

### Symptom clusters

In the symptoms models, those coughing up phlegm had higher odds of seeking help compared to those with the single symptom (coughing up phlegm) when it was reported in a cluster with shortness of breath, wheezy chest, and BJP. However, for coughing up phlegm, the odds of seeking help were lower when it was in a cluster with abdominal pain, headache and BJP. Similarly, for six other diverse symptoms (see Supplementary Table 4), the odds of help-seeking were lower when the symptom was present in a cluster. The cluster results should be interpreted with caution because the timing/temporal relationships between the symptoms within the 12-month period were unknown.

## Discussion

### Main findings and context with existing literature

Community-dwelling adults typically experienced multiple symptoms over a 12-month period. Symptom clusters usually contained non-specific symptoms such as headaches, back or join pain, and feeling tired all the time. These prevalent symptoms are known individually to contribute significantly to primary care workload and do not usually lead to a specific diagnosis [6].

The clusters give insights into the complex nature of the ‘symptom iceberg’ in the community. GP plays a key role not only in diagnosing or excluding serious illnesses from disparate groups of symptoms but also in providing holistic management at the level of the individual. There are likely to be limitations in organizing healthcare around diseases or disease clusters [18] when symptom clusters contain common symptoms such as tiredness and pain that can reduce function and quality of life [19] but are not usually associated with a specific disease.

The nature of the symptoms experienced had an important influence on GP help-seeking. For example, most people with a breast lump sought help, whereas a minority of those with headaches did so. Among those people reporting multiple symptoms but not among those reporting a single symptom, the odds of help-seeking were increased in females, those with poor health status, and those unable to work due to illness/disability. However, it is worth highlighting that for most of the social, demographic, and economic factors investigated, there was no difference between the odds of help-seeking for those presenting single and multiple symptoms. It seems likely that factors relating to individual symptoms, such as intensity, persistence, and impact on the individual are more important drivers of help-seeking than the number of symptoms experienced.

Factors associated with help-seeking varied among the different symptoms. For example, females were less likely to seek help for headache, chest pain, persistent diarrhoea, shortness of breath, blood in stool/rectal bleeding, and BJP than men, but more likely for unexplained weight loss and change in bladder habits. There is increasing recognition that the traditional stereotypes of women seeking help more readily than men are incorrect [20]. MacLean *et al.* [21] found that men’s and women’s accounts of responses to lung cancer symptoms were ‘strikingly similar’. Interestingly, for acute coronary syndrome, which often presents with chest pain, there is good evidence of delayed presentation by women compared to men [22]. Both biological and sociocultural factors have been implicated [23].

Increasing age did not have a prominent effect on help-seeking for most symptoms except headache (reduced odds of help-seeking), persistent diarrhoea, and BJP (both increased odds of help-seeking). Increasing age is an important risk factor for cancer and other diseases, so older age might have been expected to increase the odds of help-seeking across many symptoms. Our sample only included adults aged 50 and over. Nevertheless, barriers have been identified for older adults accessing primary care, and older individuals may underestimate their own risk of serious disease when appraising symptoms [24]. The influence of age on symptom appraisal, help-seeking, and the potential for diagnostic delays in older adults is an important avenue for further enquiry.

We found that smokers were less likely to seek help for some respiratory symptoms. This is in line with previous reports [25] and qualitative studies show that normalization or misattribution or respiratory symptoms, shame, stigma, and fatalism are barriers to GP help-seeking in smokers [26].

Poorer general health status, the presence of comorbidities, being unable to work, and having lower household income increased the odds of help-seeking for many different symptoms. This is in keeping with established research, which has shown that there is a ‘steep social gradient’ of increasing health needs and multimorbidity in more deprived communities [27, 28]. This places higher pressure on health services in certain geographical areas. The workload in GP is affected not only by ageing populations and multimorbidity but by wider determinants of health and the changing societal environment in which primary care functions.

### Methodological considerations

This study gives unique and granular insights into interactions between symptoms, symptom clusters, sociodemographic factors, and help-seeking in community-dwelling adults. Most literature examining symptom clusters has focused on specific diseases, such as diabetes [29]. This study has captured a clinically unselected population from the community. Participants are included who have decided not to present their symptoms to primary care. These individuals would not be captured in analyses of routinely collected health data or in retrospective, disease-focused studies.

The study used secondary data analysis and was limited by aspects of the original dataset. The response rate to the original questionnaire was relatively low (37.4%) [13]. Ethnicity was asked about in the original survey but was very poorly reported such that most data were missing, and ethnicity could not be included in analyses. Certain groups, particularly the more deprived, were under-represented [13]. This is a common limitation in questionnaire studies, and it would be important to examine whether the same symptom clusters and factors affecting help-seeking exist in other datasets and study populations.

The focus of the USEFUL questionnaire was on potential cancer symptoms. Some common physical and mental health symptoms were not asked about and the symptoms that were asked about might be weighted towards potential indicators of diagnosable physical illnesses (such as cancer). This might limit generalizability, and it will be important to extend our analyses to datasets that include psychological symptoms.

The USEFUL questionnaire relied on participant self-report about their symptoms and help-seeking and could be subject to recall bias. However, in the original study, a sub-study corroborated questionnaire data with electronic health record review and found moderate agreement between GP health records and questionnaire reports (kappa = 0.498) [30].

There is no information about the timing of symptoms within the 1-year period, whether the symptoms identified in clusters occurred simultaneously, or in which order. We studied associations, not causality, and some associations could have arisen by chance.

## Conclusions

GP deals with complexity in terms of individuals experiencing multiple disparate symptoms within a single year, and the prominent health, economic, and social influences that drive symptom consultations. Demographic factors appear to interact with symptom-related factors to modify help-seeking response so that age and sex could have a different effect on help-seeking depending on the symptom experienced. Certain groups such as single people, those with lower social contact, and smokers, may put off seeking help for certain symptoms. Those with comorbidities, poorer health, and those unable to work are more likely to seek help. GP workload will be influenced by these wider determinants of health.

## Supplementary Material

ckae198_Supplementary_Data

## Data Availability

The USEFUL study did not receive ethics approval, or participant consent, to place a study dataset in the public domain. The data used in this study can be made accessible to qualified researchers upon reasonable request pursuant to any restrictions required to ensure the privacy of human subjects involved. Access to data will be subject to a data-sharing agreement approved by the University of Aberdeen. Researchers interested in accessing USEFUL data should send their request to the study’s PI, Professor Philip Hannaford (p.hannaford@abdn.ac.uk). Key points Community-dwelling adults experience multiple disparate symptoms within a single year Help-seeking responses vary by the symptom experienced and are associated with health, economic, and social factors Certain groups such as smokers and those with lower social contact may be at risk of delayed presentation for certain symptoms Poorer health status and multimorbidity may increase help-seeking for many symptoms, and this could influence GP workload
