# Supplementary material for: Relationship between symptoms, sociodemographic factors, and general practice help-seeking in 10 904 adults aged 50 and over
Source: Eur J Public Health. 2024 Dec 15;35(1):26–34. doi: 10.1093/eurpub/ckae198 (PMC11832149; doi:10.1093/eurpub/ckae198)
Supplement: ckae198_Supplementary_Data [file ckae198_supplementary_data.zip › ckae198_Supplementary_Data/ejph-2024-06-om-0371-File002.docx]

**Supplementary file Figure S1****: study flow chart detailing questionnaire respondents and inclusion in analyses**

Questionnaire sent to 50,000 adults age ≥ 50 years

2,517 reported a symptom but did not provide information about GP help-seeking information

10,904 (65%) respondents included in analysis

3,357 reported having no symptoms

16,788 responses (33.6%)

33,212 did not respond

13,421 (80%) respondents reported at least one symptom

7638 (out of 10,905, 70%) had more than one symptom and included in cluster analysis

733 (out of 7638, 9.6%) experienced back or joint pain and headache together and were clustered a priori
