# Supplementary material for: Relationship between symptoms, sociodemographic factors, and general practice help-seeking in 10 904 adults aged 50 and over
Source: Eur J Public Health. 2024 Dec 15;35(1):26–34. doi: 10.1093/eurpub/ckae198 (PMC11832149; doi:10.1093/eurpub/ckae198)
Supplement: ckae198_Supplementary_Data [file ckae198_supplementary_data.zip › ckae198_Supplementary_Data/ejph-2024-06-om-0371-File003.docx]

**Approach to cluster analysis**

Cluster analysis is a type of multivariate analysis that groups or classifies subjects based on a set of measured variables (in this specific case, reported symptoms in the past 12 months), placing similar subjects in the same group. It is an exploratory technique which can uncover hidden structure and patterns in data.

Partitioning cluster methods (1) were used to identify distinct clusters from symptoms experienced during the previous year. These methods are more suitable to large datasets with a larger number of variables than hierarchical clustering. All the respondents experiencing more than one symptom were included in the cluster analysis, except headaches and joint/back pain, which were clustered a priori as the most common symptoms occurring alone and together. Respondents were grouped according to presence/absence (i.e. reported having experienced/ not experienced the symptom during the previous year) for all 25 symptoms. Jaccard distance was used to measure dissimilarity and clustering was achieved by applying the Partitioning around Medoids (PAM) algorithm, since this is more robust than other methods (e.g. k-Means) in the presence of noise and outliers (2,3). The Silhouette method (4) was applied as an internal validation metric to select the optimal number of clusters in our dataset, i.e. the number of clusters that yielded the highest silhouette value. The clusters were interpreted using descriptive statistics and the dimension reduction technique *t-distributed stochastic neighbourhood embedding* (t-SNE) was used to visualise clusters (5). These analyses were performed using the *cluster, Rtsne* and *ggplot2* packages in R version 3.4.1 (6–8). Results from cluster methods are sensitive to the order in which symptoms are introduced. To assess this, we conducted sensitivity analyses by varying symptom order (data not shown). The identified clusters were the same regardless of symptom order.

1. Neuhaus JM, McCulloch CE. Separating Between- and Within-Cluster Covariate Effects by Using Conditional and Partitioning Methods. J R Stat Soc Series B Stat Methodol. 2006 Nov 1;68(5):859–72.

2. Jaccard P. Etude comparative de la distribution florale dans une portion des Alpes et des Jura.

3. Kaufman L, Rousseeuw P. Clustering by means of medoids. 1987;

4. Rousseeuw PJ. Silhouettes: A graphical aid to the interpretation and validation of cluster analysis. J Comput Appl Math. 1987 Nov 1;20(C):53–65.

5. Maaten L van der, Hinton G. Visualizing Data using t-SNE. Journal of Machine Learning Research [Internet]. 2008 [cited 2023 Aug 2];9(86):2579–605. Available from: http://jmlr.org/papers/v9/vandermaaten08a.html

6. R Core Team. R: A Language and Environment for Statistical Computing. Vienna, Austria: R Foundation for Statistical Computing; 2022.

7. Krijthe JH. Rtsne: T-distributed stochastic neighbor embedding using Barnes-Hut implementation. 2015.

8. Maechler MP, Rousseeuw A, Struyf M, Hubert M, Hornik K. Cluster: Cluster analysis basics and extensions. R package version 2.0. 5; 2016. 2016.
