# Supplementary material for: Relationship between symptoms, sociodemographic factors, and general practice help-seeking in 10 904 adults aged 50 and over
Source: Eur J Public Health. 2024 Dec 15;35(1):26–34. doi: 10.1093/eurpub/ckae198 (PMC11832149; doi:10.1093/eurpub/ckae198)
Supplement: ckae198_Supplementary_Data [file ckae198_supplementary_data.zip › ckae198_Supplementary_Data/ejph-2024-06-om-0371-File004.docx]

**Supplementary data file Table S3: Proportions of participants reporting single or multiple symptoms within the preceding 12 months**

| Symptom | Number reporting this symptom | Median number of symptoms (InterQuartile Range) | Range | 1 symptom (stated symptom alone) | 2 symptoms | 3 symptoms | 4+ symptoms |
| --- | --- | --- | --- | --- | --- | --- | --- |
| ***Upper gastrointestinal symptom*** |  |  |  |  |  |  |  |
| Persistent indigestion/heartburn | 2072 | 4 (3,6) | 1,16 | 161 (7.8%) | 338 (16.3%) | 365 (17.6%) | 1208 (58.3%) |
| Difficulty swallowing | 606 | 5 (4,8) | 1,18 | 21 (3.5%) | 61 (10.1%) | 69 (11.4%) | 455 (75.1%) |
| Stomach or abdominal pain | 2032 | 5 (3,7) | 1,18 | 105 (5.1%) | 258 (12.7%) | 332 (16.3%) | 1337 (56.0%) |
| Persistent vomiting | 48 | 7 (4,8.25) | 1,14 | 2 (4.2%) | 4 (8.3%) | 2 (4.2%) | 40 (83.3%) |
| Vomiting up blood | 6 | 9 (6.5,10) | 6,10 | 0 (0.0%) | 0 (0%) | 0 (0%) | 6 (100%) |
| ***Respiratory or cardiovascular symptom*** |  |  |  |  |  |  | 0 |
| Chest pain | 1014 | 5 (3,7) | 1,18 | 42 (4.1%) | 100 (9.9%) | 126 (12.4%) | 746 (73.6%) |
| Hoarseness | 921 | 5 (4,7) | 1,18 | 26 (2.8%) | 73 (7.9%) | 131 (14.2) | 691 (75.0%) |
| Persistent cough | 1526 | 4 (3,6) | 1,18 | 109 (7.14%) | 224 (14.7) | 260 (17.0%) | 933 (61.1%) |
| Change in ongoing cough | 147 | 6 (4,9.5) | 1,18 | 3 (2.0%) | 4 (2.7%) | 22 (15.0%) | 118 (80.3%) |
| Coughing up phlegm | 1599 | 4 (3,6) | 1,18 | 86 (5.4%) | 232 (14.5%) | 289 (18.1%) | 992 (62.0%) |
| Coughing up blood | 50 | 6 (3,8) | 1,18 | 2 (4.0%) | 5 (10.0%) | 8 (16%) | 35 (70.0%) |
| Shortness of breath | 1823 | 5 (3,7) | 1,18 | 91 (5.0%) | 226 (12.4%) | 299 (16.4%) | 1207 (66.2%) |
| Wheezy chest | 1206 | 5 (3,7) | 1,18 | 48 (4.0%) | 150 (12.4%) | 182 (15.1%) | 826 (68.5%) |
| ***Colorectal symptom*** |  |  |  |  |  |  |  |
| Persistent diarrhoea | 489 | 5 (3,8) | 1,18 | 24 (4.9%) | 41 (8.4%) | 64 (13.1%) | 360 (73.6%) |
| Persistent constipation | 644 | 5 (3,7) | 1,18 | 33 (5.1%) | 75 (11.6%) | 110 (17.1%) | 426 (66.2%) |
| Change in bowel habits | 854 | 5 (3,7) | 1,18 | 45 (5.3%) | 92 (10.8%) | 151 (17.7%) | 566 (66.3%) |
| Blood in stool or rectal bleeding | 656 | 4 (2,6) | 1,18 | 59 (9.0%) | 121 (18.4%) | 111 (16.9%) | 365 (55.6%) |
| ***Breast symptom*** |  |  |  |  |  |  | 0 |
| Lump in breast | 113 | 4 (2,6) | 1,18 | 18 (15.9%) | 12 (10.6%) | 14 (12.4%) | 69 (61.1%) |
| Breast change other than lump | 139 | 4 (2,7) | 1,17 | 14 (10.1%) | 24 (17.3%) | 21 (15.1%) | 80 (57.6%) |
| ***Other symptoms*** |  |  |  |  |  |  |  |
| Loss of appetite | 582 | 6 (4,8) | 1,17 | 13 (2.2%) | 39 (6.7%) | 81 (13.9%) | 449 (77.1%) |
| Unexplained weight loss | 199 | 5 (3,7) | 1,15 | 3 (1.5%) | 21 (10.6%) | 32 (16.1%) | 143 (71.9%) |
| Tired all the time | 2155 | 5 (3,7) | 1,18 | 84 (3.9%) | 265 (12.3%) | 375 (17.4%) | 1431 (66.4%) |
| Headaches | 4997 | 3 (2,5) | 1,18 | 614 (32.3%) | 1231 (24.6%) | 997 (20.0%) | 2155 (43.1%) |
| Change in bladder habits | 1219 | 4 (2,6) | 1,18 | 141 (11.6%) | 194 (15.9%) | 218 (17.9%) | 666 (54.6%) |
| Back or joint pain | 6809 | 3 (2,4) | 1,18 | 1522 (22.4%) | 1594 (23.4%) | 1204 (17.7%) | 2489 (36.6%) |
