# Supplementary material for: Relationship between symptoms, sociodemographic factors, and general practice help-seeking in 10 904 adults aged 50 and over
Source: Eur J Public Health. 2024 Dec 15;35(1):26–34. doi: 10.1093/eurpub/ckae198 (PMC11832149; doi:10.1093/eurpub/ckae198)
Supplement: ckae198_Supplementary_Data [file ckae198_supplementary_data.zip › ckae198_Supplementary_Data/ejph-2024-06-om-0371-File005.docx]

Supplementary data file Table S4: Odds of GP help seeking for single and multiple symptoms, new and pre-existing symptoms across a range of sociodemographic factors. The table shows Odds Ratios with 95% confidence intervals.

|  | **Univariable Analysis** | | | | **Multivariable Analysis (for factors statistically significant in univariate)** | | | |
| --- | --- | --- | --- | --- | --- | --- | --- | --- |
| **Sociodemographic factor** | **Odds of seeking help: single symptom*** | **Odds of seeking help: multiple symptoms*** | **Odds of seeking help, new symptom^$^** | **Odds of seeking help, pre-existing symptom^$^** | **Odds of seeking help: single symptom*** | **Odds of seeking help: multiple symptoms*** | **Odds of seeking help, new symptom^$^** | **Odds of seeking help, pre-existing symptom^$^** |
| Male sex | 1 | 1 | 1 | 1 | 1 | 1 | 1 | 1 |
| Female sex | 1.06 (0.92-1.21) | 1.35 (1.22-1.50) | 0.97 (0.83-1.14) | 1.28 (1.18-1.38) | - | 1.40 (1.24-1.57) | - | 1.04 (0.87-1.26) |
| Age group 50-59 | 1 | 1 | 1 | 1 | 1 | 1 | 1 | 1 |
| Age group 60-69 | 1.26 (1.07-1.49) | 1.32 (1.17-1.48) | 0.98 (0.98-0.81) | 1.22 (1.11-1.34) | 1.17 (0.89-1.54) | 1.15 (0.98-1.36) | 1.09 (0.956-1.24) | 0.73 (0.56-0.94) |
| Age group 70-79 | 1.77 (1.46-2.14) | 1.50 (1.30-1.74) | 0.99 (0.80-1.23) | 1.52 (1.36-1.71) | 1.49 (1.05-2.11) | 1.18 (0.95-1.47) | 1.21 (1.02-1.44) | 0.57 (0.41-0.80) |
| Age group 80+ | 2.20 (1.63-3.00) | 2.51 (1.95-3.26) | 1.65 (1.22-2.22) | 2.55 (2.08-3.15) | 1.63 (1.01-2.65) | 1.65 (1.19-2.32) | 1.59 (1.21-2.08) | 0.96 (0.62-1.47) |
| Marital status: Married/living together | 1 | 1 | 1 | 1 | 1 | 1 | 1 | 1 |
| Marital status: Single | 0.81 (0.61- 1.08) | 0.93 (0.76-1.14) | 0.94 (0.68-1.28) | 0.92 (0.78-1.09) | - | 0.78 (0.62-0.98) | 0.78 (0.64-0.94) | 0.75 (0.52-1.06) |
| No longer married | 1.17 (0.97 –1.41) | 1.27 (1.11-1.46) | 1.23 (1.00-1.50) | 1.35 (1.21-1.51) | - | 0.95 (0.81-1.12) | 1.07 (0.94-1.22) | 1.05 (0.83-1.34) |
| Health status: Excellent | 1 | 1 | 1 | 1 | 1 | 1 | 1 | 1 |
| Health status: Very good | 1.29 (1.05-1.60) | 1.71 (1.38-2.12) | 1.45 (1.14-1.86) | 1.83 (1.57-2.13) | 1.43 (1.06-1.95) | 1.47 (1.17-1.86) | 1.64 (1.39-1.94) | 1.38 (1.06-1.80) |
| Health status: Good | 1.97 (1.58-2.45) | 2.18 (1.77-2.69) | 1.95 (1.51-2.52) | 2.89 (2.48-3.37) | 2.17 (1.58-3.00) | 1.57 (1.25-1.99) | 2.18 (1.84-2.58) | 1.58 (1.19-2.15) |
| Health status: Fair | 3.52 (2.53-4.92) | 4.54 (3.57-5.78) | 4.01 (2.85-5.65) | 6.54 (5.43-7.89) | 2.41 (1.55-3.78) | 2.78 (2.12-3.64) | 3.96 (3.21-4.89) | 2.78 (1.86-4.16 |
| Health status: Poor | 2.68 (1.14-6.62) | 10.80 (7.02-17.21) | 4.24 (18.71) | 15.15 (10.40-22.78) | 1.96 (0.71-5.38) | 5.31 (3.23-9.05) | 6.42 (4.23-10.04) | 5.81 (2.48-13.55) |
| Comorbid conditions: None | 1 | 1 | 1 | 1 | 1 | 1 | 1 | 1 |
| Comorbid conditions:  1 or 2 | 1.67 (1.42-1.97) | 1.88 (1.65-2.15) | 1.61 (1.34-1.94) | 1.97 (1.78-2.19) | 1.61 (1.27-2.06) | 1.70 (1.46-1.98) | 1.68 (1.49-1.89) | 1.44 (1.16-1.78) |
| Comorbid conditions:  3 or more | 2.71 (2.15-3.42) | 3.87 (3.31-4.54) | 2.67 (2.07-3.45) | 4.61 (4.05-5.24) | 2.06 (1.47-2.87) | 2.78 (2.30-3.36) | 2.99 (2.56-3.49) | 1.94 (1.42-2.64) |
| Never smoked | 1 | 1 | 1 | 1 | 1 | 1 | 1 | 1 |
| Ex-smoker | 1.13 (0.98-1.31) | 1.23 (1.10-1.37) | 1.16 (0.98-1.38) | 1.27 (1.16-1.38) | - | 1.05 (0.93-1.19) | - | 1.02 (0.84-1.23) |
| Current smoker | 1.04 (0.78-1.37) | 1.08 (0.90-1.30) | 1.05 (0.77-1.41) | 1.21 (1.04-1.41) | - | 0.90 (0.73-1.10_ | - | 0.76 (0.54-1.07) |
| Working full-time | 1 | 1 | 1 | 1 | 1 | 1 | 1 | 1 |
| Working part-time | 1.15 (0.89-1.50) | 1.32 (1.09-1.61) | 1.05 (0.75-1.45) | 1.22 (1.05-1.42) | 0.97 (0.68-1.39) | 1.09 (0.88-1.35) | 1.14 (0.96-1.35) | 1.02 (0.71-1.44) |
| Self-employed | 0.89 (0.66-1.20) | 1.08 (0.89-1.32) | 1.31 (0.94-1.80) | 1.03 (0.88-1.22) | 0.96 (0.63-1.45) | 1.00 (0.81-1.25) | 0.95 (0.79-1.14) | 1.36 (0.95-1.93) |
| Retired | 1.39 (1.17-1.66) | 1.51 (1.34-1.71) | 1.27 (1.04-1.56) | 1.40 (1.27-1.55) | 0.86 (0.62-1.18) | 0.95 (0.78-1.14) | 0.92 (0.79-1.07) | 1.12 (0.83-1.52) |
| Unable to work due to illness/disability | 1.48 (0.60-3.66) | 4.88 (3.29-7.52) | 4.18 (1.85-9.10) | 6.07 (4.18-8.94) | 0.91 (0.35-2.37_ | 1.83 (1.15-3.01) | 1.99 (1.31-3.13) | 1.59 (0.61-3.92) |
| Others not in paid employment | 1.21 (0.79-1.85) | 1.72 (1.29 – 2.33) | 1.35 (0.81-2.17) | 1.52 (1.21-1.92) | 0.93 (0.51-1.69) | 1.37 (0.98-1.93) | 1.22 (0.94-1.59) | 1.26 (0.71-2.15) |
| Household income >£50,0000 | 1 | 1 | 1 | 1 | 1 | 1 | 1 | 1 |
| Household income < £15,000 | 1.62 (1.30-2.03) | 1.95 (1.66-2.29) | 1.84 (1.42-2.38) | 1.89 (1.66-2.15) | 1.10 (0.79-1.54) | 1.16 (0.94-1.43) | 1.15 (0.98-1.35) | 1.81 (1.29-2.54) |
| Household income £15,000-29,999 | 1.30 (1.06-1.59) | 1.62 (1.40-1.88) | 1.49 (1.16-1.91) | 1.42 (1.26-1.60) | 0.95 (0.71-1.27) | 1.20 (1.01-1.43) | 1.07 (0.93-1.22) | 1.49 (1.12-1.98) |
| Household income £30,000-49,999 | 1.19 (0.96-1.47) | 1.32 (1.13-1.54) | 1.24 (0.95-1.62) | 1.24 (1.09-1.39) | 1.01 (0.76-1.35) | 1.16 (0.99-1.37) | 1.09 (0.95-1.24) | 1.25 (0.94-1.65) |
| Degree or postgraduate qualifications | 1 | 1 | 1 | 1 | 1 | 1 | 1 | 1 |
| No educational qualifications | 1.48 (1.16-1.90) | 1.79 (1.48-2.18) | 1.12 (0.85-1.46) | 1.74 (1.49-2.02) | 0.91 (0.63-1.31) | 1.16 (0.92-1.46) | - | 0.79 (0.57-1.10) |
| Secondary school or equivalent | 1.26 (1.06-1.52) | 1.42 (1.25-1.64) | 1.17 (0.95-1.45) | 1.35 (1.21-1.50) | 0.92 (0.71-1.20) | 1.14 (0.97-1.33) | - | 1.01 (0.79-1.28) |
| College/vocational courses and other | 1.06 (0.70-1.62) | 1.57 (1.20-2.08) | 1.00 (0.62-1.56) | 1.47 (1.18-1.84) | 0.94 (0.53-1.66) | 1.25 (0.92-1.71) | - | 0.74 (0.43-1.22) |
| Professional qualification | 1.05 (0.87-1.27) | 1.19 (1.04-1.37) | 1.18 (0.94-1.48) | 1.10 (0.99-1.23) | 0.82 (0.63-1.06) | 1.07 (0.92-1.25) | - | 1.04 (0.81-1.33) |
| High social contact | 1 | 1 | 1 | 1 | 1 | 1 | 1 | 1 |
| Low social contact | 0.96 (0.68-1.34) | 0.85 (0.69-1.05) | 0.98 (0.66-1.42) | 0.94 (0.79-1.12) | - | - | - | - |
| Medium social contact | 0.96 (0.83-1.12) | 0.92 (0.83-1.03) | 0.92 (0.77-1.10) | 0.94 (0.86-1.02) | - | - | - | - |
| GP registration in Scotland | 1 | 1 | 1 | 1 | 1 |  | 1 | 1 |
| GP registration in England | 1.14 (0.99-1.30) | 1.07 (0.97-1.19) | 1.07 (0.91-1.25) | 1.04 (0.96-1.13) | - | - | - | - |
| Distance to GP Under one mile | 1 | 1 | 1 | 1 | 1 | 1 | 1 | 1 |
| Distance to GP 1-5 miles | 0.95 (0.80-1.13) | 0.90 (0.80-1.01) | 0.97 (0.71-1.33) | 0.92 (0.84-1.01) | 0.90 (0.74-1.09) | - | - | - |
| Distance to GP 6-10 miles | 0.64 (0.43-0.95) | 0.86 (0.68-1.09) | 0.50 (0.24-1.06) | 0.86 (0.71-1.04) | 0.58 (0.37-0.90) | - | - | - |
| Travel time to GP <15 minutes | 1 | 1 | 1 | 1 | 1 | 1 | 1 | 1 |
| Travel time to GP 15-29 minutes | 1.10 (0.89-1.35) | 1,00 (0.87-1.14) | 0.97 (0.67-1.42) | 1.08 (0.96-1.20) | - | - | - | - |
| Travel time to GP 30-59 minutes | 0.83 (0.48-1.44) | 0.80 (0.59-1.09) | 1.35 (0.60-3.32) | 0.81 (0.62-1.05) | - | - | - | - |

**Odds of seeking help for a single symptom is for those individuals who only reported one symptom in isolation within the previous 12 months. Multiple symptoms are those who experienced more than one symptom within the previous 12 months.*

*$Odds of seeking help for a new symptom refers to those who experienced a single symptom as a new symptom within the past 12 months. Pre-existing symptoms refer to those single symptoms that were present prior to the past 12 months.*
