# Supplementary material for: Relationship between symptoms, sociodemographic factors, and general practice help-seeking in 10 904 adults aged 50 and over
Source: Eur J Public Health. 2024 Dec 15;35(1):26–34. doi: 10.1093/eurpub/ckae198 (PMC11832149; doi:10.1093/eurpub/ckae198)
Supplement: ckae198_Supplementary_Data [file ckae198_supplementary_data.zip › ckae198_Supplementary_Data/ejph-2024-06-om-0371-File006.docx]

**Supplementary data Table S5 of factors associated with the odds of seeking help from a GP for each symptom.** Only results which were statistically significant at α<0.05 are shown.

| **Symptom** | **Decreased odds of seeking help*^$^** | **Increased odds of seeking help*** |
| --- | --- | --- |
| Headaches | **Female** OR 0.42 (0.33-0.53) | **Unable to work** OR 2.99 (1.90-4.70) |
|  | **Age 60-69** OR 0.73 (0.57-0.93)  **Age 70-79** OR 0.67 (0.47-0.96) | **Household income** £15,000-£29,000 OR 1.52 (1.13-2.05)  **Household income** <£14,999 OR 1.47 (1.13-1.93) |
|  | **GP location England** OR 0.74 (0.61-0.88) | **General health good** OR 1.52 (1.05-2.24)  **General health fair** OR 2.06 (1.36-3.18)  **General health poor** OR 3.04 (1.69-5.47) |
|  | **Medium social contact** OR 0.66 (0.54-0.80) |  |
|  | **Had symptom before last year** OR 0.42 (0.33-0.52) |  |
|  | Presence in headaches and BJP **cluster** OR 0.69 (0.48-0.99)  Presence in abdominal pain, headaches, and BJP **cluster** OR 0.65 (0.45-0.95)  Presence in coughing phlegm and BJP **cluster** OR 0.32 (0.16-0.59) |  |
| Persistent Indigestion/ heartburn | **Self-employed** OR 0.65 (0.42-0.99) | **Unable to work** OR 2.09 (1.23-3.61)  **Not in paid employment** OR 1.72 (1.01-2.95) |
|  | **Current smoker** OR 0.65 (0.45-0.99) | **1 or 2 comorbidities** OR 1.78 (1.31-2.44)  **3+ comorbidities** OR 2.62 (1.86-3.73) |
| Difficulty swallowing |  | **No qualifications** OR 2.05 (1.08-3.91)  **Secondary education** OR 1.73 (1.05-2.89)  **College/vocational** OR 5.71 (2.32-15.11)  **Professional qualifications** OR 1.71 (1.02-2.91) |
| Stomach or abdominal pain | **Medium social contact** OR 0.71 (0.57-0.89) | **General health fair** OR 2.39 (1.39-4.17)  **General health poor** OR 4.81 (2.45-9.64) |
|  | Presence in abdominal pain, headaches, and BJP **cluster** OR 0.27 (0.16-0.44)  Presence in indigestion, headaches and BJP **cluster** OR 0.28 (0.15-0.51)  Presence in persistent cough and headaches **cluster** OR 0.11 (0.03-0.34)  Presence in coughing phlegm and BJP **cluster** F OR 0.31 (0.15-0.65)  Presence in varied multi-symptom **cluster** OR 0.27 (0.15-0.47)  Presence in shortness of breath, wheezy chest and BJP **cluster** OR 0.17 (0.08-0.36)  Presence in respiratory predominant multi-symptom **cluster** OR 0.18 (0.09-0.35) | **1 or 2 Comorbidities** OR 1.44 (1.05-1.98)  **3+ Comorbidities** OR 1.89 (1.32-2.72) |
| Chest pain | **Female** OR 0.56 (0.40-0.77) | **Part time employment** OR 1.82 (1.03-3.30)  **Retired** OR 1.59 (1.11-2.28) |
|  | **Medium social contact** OR 0.64 (0.47- 0.87) |  |
| Hoarseness |  | **3+ Comorbidities** OR 2.07 (1.19-3.74) |
| Loss of appetite | **Medium social contact** OR 0.62 (0.38-0.98) | **Poor general health** OR 4.80 (1.16-26.12) |
| Unexplained weight loss |  | **Female** OR 2.67 (1.19-6.49) |
|  |  | **3+ Comorbidities** OR 4.12 (1.20-15.68) |
| Persistent cough | **Current smoker** OR 0.48 (0.33-0.71) |  |
|  | Presence in abdominal pain, headaches and BJP **cluster** OR 0.43 (0.22-0.85)  Presence in indigestion, headaches and BJP **cluster** OR 0.49 (0.26-0.91)  Presence in persistent cough and headaches **cluster** OR 0.43 (0.25-0.71)  Presence in coughing phlegm and BJP **cluster** OR 0.46 (0.27-0.77)  Presence in varied multi-symptom **cluster** OR 0.29 (0.13-0.63) |  |
| Persistent diarrhoea | **Female** OR 0.58 (0.37-0.92) | **Age** 60-69 OR 1.84 (1.17-2.91)  **Age** 70-79 OR 3.41 (1.84-6.51)  **Age** 80+ OR 3.83 (1.34-12.63) |
|  |  | **Poor general health** OR 4.29 (1.18 – 17.17) |
| Persistent constipation |  | **Household income** £15000 - £29,000 OR 2.11 (1.21-3.75) |
| Coughing up phlegm | **Current smoker**  OR 0.56 (0.37-0.84) | Presence in shortness of breath, wheezy chest and BJP **cluster** OR 2.28 (1.16-4.58) |
|  | Presence in abdominal pain, headache and BJP cluster OR 0.28 (0.15-0.65) |  |
| Shortness of breath | **Female** OR 0.68 (0.51–0.89) | **Household income** <£14,999 OR 1.49 (1.03-2.17) |
|  | **Marital status single** OR 0.52 (0.34-0.79) | **General health fair** OR 2.19 (1.04-4.67)  **General health poor** OR 2.72 (1.20-6.24) |
|  | **Current smoker** OR 0.59 (0.40-0.87) | **Comorbidities 3+** OR 2.16 (1.28-3.70) |
|  | **Medium social contact** OR 0.77 (0.61-0.98) |  |
|  | Presence in abdominal pain, headaches, and BJP **cluster** OR 0.23 (0.10-0.53)  Presence in indigestion, headaches and BJP **cluster** OR 0.28 (0.14-0.53)  Presence in cough and headaches **cluster** OR 0.41 (0.20-0.81)  Presence in tiredness, headaches, and BJP **cluster** OR 0.18 (0.08-0.39)  Presence in coughing phlegm and BJP **cluster** OR 0.42 (0.21-0.81)  Presence in varied multi-symptom **cluster** OR 0.34 (0.18-0.62)  Presence bladder symptom and BJP **cluster** 0.26 (0.11-0.59) |  |
| Wheezy Chest | **Medium social contact** OR 0.75 (0.57-0.99) | **Unable to work** OR 3.10 (1.52 – 6.77) |
| Change in bladder habits | Presence in abdominal pain, headaches, and BJP **cluster** OR 0.42 (0.22-0.82)  Presence in indigestion, headaches and BJP **cluster** OR 0.38 (0.20-0.72)  Presence in persistent cough and headaches **cluster** OR 0.29 (0.16-0.53)  Presence in tiredness, headaches, and BJP **cluster** OR 0.25 (0.12-0.51)  Presence in coughing phlegm and BJP **cluster** OR 0.32 (0.17-0.62)  Presence in varied multi-symptom **cluster** OR 0.32 (0.17-0.61)  Presence bladder symptom and BJP **cluster** OR 0.39 (0.24-0.63)  Presence in respiratory predominant multi-symptom **cluster** OR 41 (0.20-0.85) | **Female** OR 1.36 (1.04-1.77) |
|  |  | **3+ comorbidities** OR 1.77 (1.15-2.74) |
| Change in bowel habits | **Self-employed** OR 0.49 (0.25-0.91) | **No qualifications** OR 2.05 (1.10 – 3.90)  **Secondary school qualifications** OR 1.83 (1.19-2.80) |
|  |  | **1 or 2 Comorbidities** OR 2.17 (1.32-3.64)  **3+ Comorbidities** OR 3.35 (1.92-5.93) |
| Blood in stool/ rectal bleeding | **Female** OR 0.45 (0.30-0.66) | **Unable to work** OR 3.94 (1.60-10.74) |
| Back or joint pain | **Female** OR 0.82 (0.68-0.98) | **Age 70-79** OR 1.33 (1.08-1.65) |
|  | **Low social contact** OR 0.63 (0.50-0.81)  **Medium social contact** OR 0.86 (0.76-0.97) | **Unable to work** OR 2.10 (1.44-3.10) |
|  | **Professional** **education** OR 0.83 (0.72-0.97) | **General health very good** OR 1.48 (1.17-1.87)  **General health good** OR 1.95 (1.54-2.48)  **General health fair** OR 3.05 (2.33-4.00)  **General health poor** OR 4.23 (2.83-6.38) |
|  | **GP location England** OR 0.87 (0.78-0.98) | **1 or 2 Comorbidities** OR 1.28 (1.10-1.50)  **3+ Comorbidities** OR 1.83 (1.51-2.21) |
|  | Presence in headaches and BJP **cluster** OR 0.65 (0.53-0.80)  Presence in abdominal pain, headaches, and BJP **cluster** OR 0.78 (0.63-0.96)  Presence in indigestion, headaches and BJP **cluster** OR 0.62 (0.52-0.75)  Presence in persistent cough and headaches **cluster** OR 0.44 (0.25 – 0.74)  Presence in coughing phlegm and BJP **cluster** OR 0.65 (0.53-0.79)  Presence in shortness of breath, wheezy chest and BJP **cluster** OR 0.71 (0.55-0.90)  Presence in indigestion and abdominal pain **cluster** OR 0.51 (0.30-0.84)  Presence in respiratory predominant multi-symptom **cluster** OR 0.63 (0.46-0.87) |  |
| Tired all the time | **GP location England** OR 0.61 (0.50-0.75)  **Low social contact** OR 0.63 (0.43-0.91) | **Household income** <£14,999 OR 1.54 (1.14-2.08)  **Household income** £15,000-£29,999 OR 1.35 (1.01-1.81) |
|  |  | **General health poor** OR 2.79 (1.39-5.76) |
|  |  | **1 or 2 Comorbidities** OR 1.47 (1.06-2.04)  **3+ Comorbidities** OR 1.94 (1.37-2.77) |

**Reference categories: Gender = male; Age = 50-59 years; Employment = full time; Household income = >£50,000; GP location = Scotland; Social contact = high social contact; General health = excellent; Comorbidities count = 0; had symptom before the last year = no, smoking = never smoked, marital status = married/living together, Educational Status = degree or postgraduate education; Presence in a cluster = being an isolated symptom*

*$ Independent models were conducted for each symptom (see methods) and each symptom/row should be considered as a unique analysis. The symptoms have been presented in one table for ease of presentation.*
